# A Refined Rotation Forest-Based Ensemble Classifier for Lithological Mapping with ZY1-02D Hyperspectral Remote Sensing Imagery

**DOI:** 10.3390/s26113340

**Published:** 2026-05-25

**Authors:** Jing Xi, Hailiang Gao, Wenqian Zang, Xiaofei Zhao, Miao Liu, Bin Li

**Affiliations:** 1 National Engineering Research Center of Satellite Remote Sensing Applications, Aerospace Information Research Institute, Chinese Academy of Sciences, Beijing 100094, China; xijing@aircas.ac.cn (J.X.);; 2 University of Chinese Academy of Sciences, Beijing 100049, China

**Keywords:** hyperspectral data, lithological mapping, ensemble learning, rotation forest

## Abstract

**Highlights:**

**What are the main findings?**

- The improved ROF-LightGBM model with optimized base classifiers achieves higher classification accuracy and computational efficiency than conventional rotation forest, random forest and LightGBM models. It also possesses better robustness under limited training samples and noisy data interference.
- In this study, minimum noise fraction (MNF) is introduced to construct the rotation matrix for the improved ROF-LightGBM framework. Compared with PCA and ICA, MNF is more suitable for lithological classification tasks, which further boosts the overall performance of the modified model.

**What are the implications of the main findings?**

- This study proposes an optimized ROF-LightGBM (MNF) framework to address the low computational efficiency of conventional ROF algorithms when processing high-dimensional remote sensing data.
- The method realizes effective lithological recognition in bedrock outcrops, offering solid technical support for high-precision geological investigation and mapping.

**Abstract:**

Hyperspectral remote sensing data provides distinct advantages for lithological classification in bedrock-exposed areas. Despite the superior performance of ensemble learning methods (e.g., Rotation Forest, ROF) in big data classification, their application in high-dimensional hyperspectral data is restricted by high training costs. To address this limitation and improve classification accuracy, this study proposes an optimized ROF-LightGBM ensemble algorithm integrated with minimum noise fraction (MNF) for rotation matrix construction. Experimental validation was conducted using ZY1-02D hyperspectral data for lithological mapping in the bedrock-exposed Xitieshan area, involving ROF-LightGBM parameter optimization (L × T, bootstrap) and comparative experiments with multiple machine learning models. The results demonstrate the following: under the same number of decision trees (T = 100), the ROF-LightGBM (PCA, L × T = 4 × 25) with optimized base classifier outperforms random forest (RF), LightGBM, and traditional ROF (L = 100) models in classification accuracy, achieving 74.28% accuracy, 6.54% higher than RF and 1.53% higher than LightGBM. More notably, it boasts exceptional efficiency, with a training time of only 4.86 s (nearly 37 times shorter than traditional ROF), while maintaining minimal accuracy loss (an only 1.19% decrease). Additionally, the ROF-LightGBM (MNF) model, which adopts MNF for rotation matrix construction, further enhances performance. Compared with the PCA-based ROF-LightGBM, it achieves an 82.17% classification accuracy (a 7.89% increase) and its kappa coefficient reaches 0.81, fully verifying the model’s superiority in accuracy and efficiency.

## 1. Introduction

Characterized by macro-scale, multi-angle, multi-temporal and multi-level information attributes, remote sensing data provides robust support for geological research and exploration and has become an indispensable core technical tool in geological research [1]. Traditional geological survey and mineral exploration methods are constrained by high resource consumption, long cycles, harsh conditions, strict professional requirements, and an inability to update data in real time, with limitations more pronounced in areas with complex geology and inaccessible terrain due to poor personnel reachability. By contrast, remote sensing technology overcomes topographic constraints, greatly reduces field workload, and improves survey efficiency, providing a reliable technical approach for geological surveys [2,3].

The spectral mechanisms of rocks and minerals constitute the core theoretical foundation for lithological identification employing remote sensing technology. When irradiated by electromagnetic waves across different spectral bands (0.4–2.5 μm), distinct rocks and minerals undergo electronic transitions or vibrational, thereby endowing them with unique spectral absorption features [4,5,6]. As aggregates composed of multiple mineral phases, the spectral properties of rocks are predominantly influenced by their internal mineral constituents and overall structural configurations, manifesting complex absorption bands and reflection peaks [7,8]. Hyperspectral data imaging targets via dozens or even hundreds of subdivided contiguous spectral bands, capturing abundant distinctive rock spectral information (0.4–2.5 μm) and showing great potential in lithological identification. Dadon et al. utilized Hyperion data to identify the stratigraphic lithology of Dana National Geopark, successfully distinguishing plutonic rocks (monzogranite, granite, and granodiorite), volcanic rocks (green tuff and basaltic lava), and sedimentary rocks (limestone, sandstone, conglomerate, dolomite, and shale) [9]. Kumar et al. integrated AVIRIS-NG hyperspectral data to classify the Hutti gold-mineralized granite–chlorite rock in India and obtained a high-resolution lithological classification map of the region [10]. The ZY1-02D hyperspectral satellite was launched on 12 September 2019. It carries a hyperspectral imager (HSI) with two imaging sensors operating in the visible to near-infrared (VNIR) and shortwave infrared (SWIR) spectral regions (http://www.sasclouds.com/english/, accessed on 16 March 2022). With a swath width of 60 km and a spatial resolution of 30 m, the satellite has achieved favorable performance in lithological mapping, allowing large-area coverage with fewer scenes. Lin et al. carried out lithological classification research in the Lenghu area at the junction of Qinghai Province and Gansu Province using ZY1-02D data, and the lithological classification results were basically consistent with field verification [11]. Yu et al. used ZY1-02D data to conduct lithological identification research in Liuyuan Town, Gansu Province [12]. While hyperspectral imagery provides abundant spectral information for remote sensing lithological identification, it has inherent limitations [13,14]. For instance, available hyperspectral data is scarce, restricted to fixed global regions; despite simultaneous operation of multiple hyperspectral sensors, their quantity is insufficient. Additionally, redundant information in hyperspectral data induces the “Huge” phenomenon during lithological identification.

With the rapid advancement of computer science and technology, remote-sensing-based lithology identification has undergone a paradigm shift from traditional manual visual interpretation to automated classification driven by artificial intelligence (AI) techniques. Currently, data-driven intelligent algorithms, including machine learning (ML) and deep learning (DL) approaches, have demonstrated tremendous potential in processing hyperspectral datasets and extracting valuable geological information embedded within such high-dimensional data. Python 3.8, ArcGIS 10.8, ENVI 5.6 et al. are mainstream tools for algorithm modeling and remote sensing data analysis in relevant research. Commonly adopted ML-based methodologies in this research domain include support vector machines (SVM) [15], k-nearest neighbors (KNN) [16], decision trees (DTs) [17], random forests (RFs) [18], and back-propagation (BP) neural networks [19], as well as linear or logistic regression [20]. However, traditional ML methods are inherently constrained when addressing high-dimensional hyperspectral tasks, characterized by challenges such as small sample sizes and class imbalance. These inherent limitations frequently lead to the curse of dimensionality and overfitting, which subsequently deteriorate the overall performance of lithology classification models.

In recent years, DL techniques like convolutional neural networks (CNNs), transformer architectures, and spectral–spatial hybrid networks have attained remarkable achievements in hyperspectral lithology classification. Santos V S D et al. developed SCB-Net to address geological mapping limitations, achieving higher lithological mapping accuracy than Attention U-Net [21]. Dong Y et al. fused GF-5 and Sentinel-2B images via an SFIM strategy and proposed ViT-DGCN, which achieves superior lithological mapping performance with scarce training samples [22]. Li C et al. proposed a deep neural network framework with multi-shared feature fusion layers oriented to geological routes to realize intelligent regional geological mapping, which fully excavates multimodal data features and outperforms traditional and random-forest-based mapping methods [23]. Such DL-based approaches possess robust capabilities in extracting discriminative spatial–spectral features from geological big data, thereby providing effective technical support for high-precision lithological mapping. By enabling hierarchical and adaptive feature learning, these models can directly abstract meaningful spectral–spatial patterns from raw hyperspectral imagery, eliminating the need for labor-intensive manual feature engineering. Despite their superior classification performance, DL-based hyperspectral image (HSI) classification still encounters several prominent challenges, including high model complexity, excessive computational latency, and a heavy dependence on a sufficient volume of high-quality labeled ground truth samples [24]. Furthermore, the majority of existing DL-based lithology classification methods adopt a patch-based framework, which is plagued by critical drawbacks: spatial discontinuity in classification outputs induced by inadequate contextual information modeling and low inference efficiency resulting from redundant computations in overlapping image patches. These limitations significantly restrict the geospatial coherence and scalability of classification results, particularly in the context of large-scale geological remote sensing applications [25].

As a key focus in machine learning, ensemble learning optimizes classification results and mitigates overfitting via a unified data fusion, modeling, and mining framework [26]. Its core strategies, Bagging and Boosting [27], respectively excel in reducing model bias and enhancing base classifier diversity. The gradient boosting algorithm, proposed by Friedman [28], is a representative Boosting method, with XGBoost as its classic variant. XGBoost integrates feature importance evaluation and feature-level parallel computing to improve the training efficiency of large datasets, regulates model complexity via regularization, and supports targeted missing value handling, thereby enhancing generalization ability while suppressing overfitting [29]. Nevertheless, XGBoost still suffers from high time complexity and limited optimization efficiency for large-scale high-dimensional datasets, despite mitigating the inefficiency of GBDT via node-splitting pre-sorting [30]. To address this challenge, Ke et al. proposed LightGBM [27,31], which retains many advantages of XGBoost, such as parallel training, regularization, and sparse optimization. The major difference between the two lies in the method of constructing trees: LightGBM adopts a leafwise split strategy, where after the first split, the node with a higher delta loss is selected for subsequent splitting. This technique enables LightGBM to easily handle huge amounts of data. Additionally, LightGBM employs a histogram-based feature discretization and optimal split strategy; by mapping continuous features to limited-interval histograms, it significantly reduces the computational overhead of feature traversal and splitting, lowers memory usage while ensuring accuracy, and concurrently improves generalization ability and training efficiency.

Bagging generates sub-datasets via bootstrapping, constructs base classifiers on each dataset, and fuses results via mean, median, or voting [32]. However, the similarity of sub-samples limits the diversity of base classifiers and the performance of the ensemble model. As a Bagging variant, RF enhances diversity by randomly selecting features during node splitting without significant accuracy loss and is widely used in remote sensing classification due to its low computational complexity, excellent classification performance, and anti-overfitting capability in high-dimensional data [33]. Based on the idea that random injection can increase diversity, Rodriguez et al. proposed ROF based on RF [34], which transforms the feature space via PCA to generate distinct subsets for modeling and outperforms RF on multiple benchmarks. Ensemble learning theory [35] indicates that ensemble generalization correlates with the average error and diversity of base classifiers, where smaller error and higher diversity improve performance. ROF has been proven to outperform other ensemble approaches significantly on several benchmark classification datasets, including unbalanced datasets, hyperspectral image datasets, and gene microarray datasets. While it achieves high classification accuracy, this superior performance comes at the cost of increased training time. Notably, research regarding the application of this method in lithological classification is limited, and few relevant empirical studies have been carried out thus far.

Extensive endeavors have been undertaken to refine the ROF algorithm, with improvements primarily focusing on four key dimensions: the integration of ROF with other ensemble learning methods, the replacement of principal component analysis (PCA) with alternative feature extraction mechanisms, the modification of base classifier types, and the introduction of novel feature variables et al. [36]. For instance, Zhang et al. [37] integrated AdaBoost with ROF to develop the RotBoost algorithm, which was shown to outperform both standalone ROF and AdaBoost in terms of prediction error. Tan et al. proposed an Improved Adaptive Boosting Rotation Forest (IABROF) algorithm for air quality prediction, reporting an approximate 10% increase in average test set accuracy [38]. Additionally, Gu et al. presented a novel ensemble classifier named RoF-GBM [39], which was rigorously evaluated across 12 small-to-medium-sized datasets, three large datasets, three high-dimensional datasets, and two artificial datasets. Their results verified that RoF-GBM effectively enhances both classification accuracy and efficiency relative to the original ROF. However, despite the proliferation of these modified ROF variants, most existing improvements focus primarily on enhancing classification accuracy, while the issue of classification efficiency remains poorly addressed. In addition, their application specifically to the domain of hyperspectral lithological classification has not been investigated to date.

This study addresses the key limitation of traditional ROF models in hyperspectral lithology classification, namely low computational efficiency when processing high-dimensional spectral data. Taking bedrock outcrops in Xitieshan, Qinghai Province as the research area, this paper constructs an optimized ROF-LightGBM lithology classification model. The core highlight and primary contribution of this research lie in the targeted improvement of the original rotation forest framework to adapt to geological spectral identification demands. The proposed method inherits the basic structure of classic ROF and adopts two effective improvement strategies. Firstly, LightGBM is used to replace the original base decision tree classifier to strengthen overall prediction performance. Secondly, MNF transformation is utilized to build the rotation matrix of feature space. This design effectively enlarges the feature difference and sample diversity of different lithologies and greatly improves the recognition capability for subtle spectral features difficult to distinguish in original hyperspectral data. Combined with quantitative index evaluation and field verification results, the proposed ROF-LightGBM model achieves prominent improvements in both classification accuracy and operational efficiency for hyperspectral lithology mapping.

This paper is organized as follows. Section 1 reviews the relevant literature and research progress. Section 2 describes the study area and geological background in detail. Section 3 elaborates on the data processing workflow and the optimized ROF-LightGBM model. Section 4 presents the experimental results and analysis. Section 5 discusses the research findings. Finally, Section 6 summarizes the conclusions and future prospects.

## 2. Geological Background

**Figure 1:**
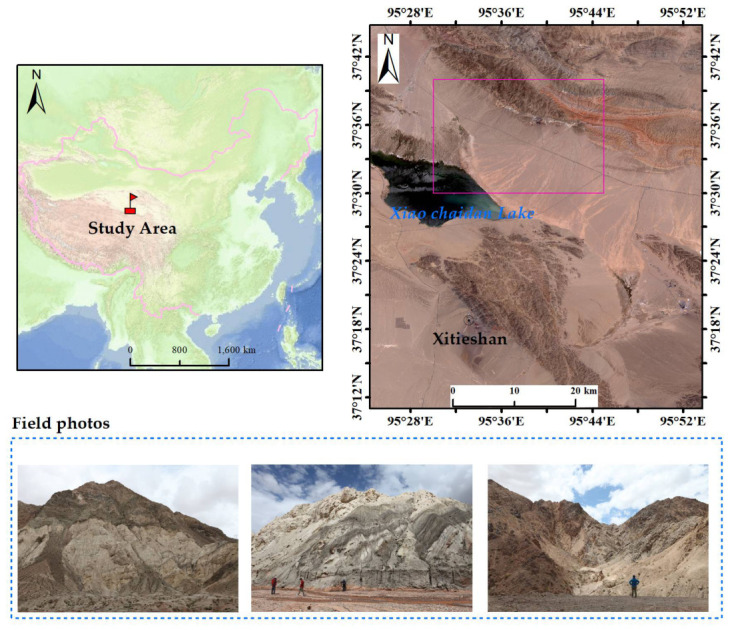
Location map and field photos of the Xitieshan study area.

The study area is located on the northern margin of the Qaidam Basin in the northeastern part of the Qinghai–Tibet Plateau, belonging to the Northern Qaidam Tectonic Belt (Figure 1). This area is bordered by the Qilian Block to the north and the Qaidam Block to the south, and is divided into the Oulongbuluke Block and the Yukahe Ultra-High Pressure Belt by the Yuka–Wulan Fault [40,41]. The ultra-high pressure metamorphic belt extends approximately 400 km in the NWW direction, covering the Yuka, Lüliangshan, Xitieshan, and Dulan terranes. The Northern Qaidam has experienced complex orogenic processes during the Caledonian, Hercynian, and Indosinian periods, and is an important metallogenic belt in western China, where the Xitieshan super-large lead–zinc deposit and the Tanjianshan large gold deposit have been discovered. The basement of this area is mainly composed of gneiss, basic granulite, marble, amphibolite, and locally eclogite and garnet peridotite. It is divided into the Yuka Terrane, Lüliangshan Terrane, Xitieshan Terrane, and Dulan Terrane, with predominant outcrops of granitic and argillaceous gneiss, and locally contains eclogite lenses and interlayers [42,43,44].

**Figure 2:**
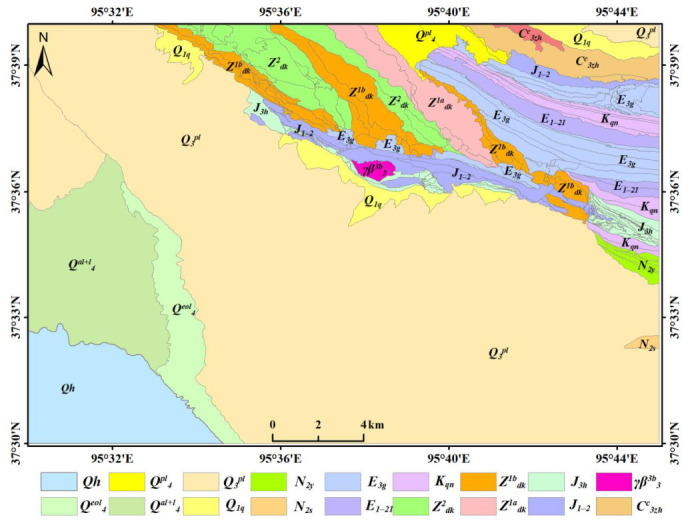
Geological map of the Xitieshan study area.

Lithological mapping for the bedrock-exposed Xitieshan area was performed based on the 1:200,000 geological map of the study area, integrated with GF-1/GF-2 satellite imagery, shown in Figure 2. A comprehensive set of remote sensing image processing techniques, including band ratioing, principal component analysis (PCA), and spatial color transformation enhancement, were employed, and the interpretation results were further verified and revised through field reconnaissance surveys. The Xitieshan study area comprises 19 stratigraphic lithologic units in total, sequentially distributed from the oldest to the youngest chronologically as follows: the Paleoproterozoic Dakendaban Group, the Cambrian–Ordovician Tanjianshan Group, the Early Carboniferous Chengqianggou Formation, the Early–Middle Jurassic Dameigou Formation, the Oligocene–Miocene Ganchaigou Formation, the Miocene Youshashan Formation, the Early Pleistocene Qiigequan Formation, and quaternary deposits of diverse genetic types.

## 3. Materials and Methods

**Figure 3:**
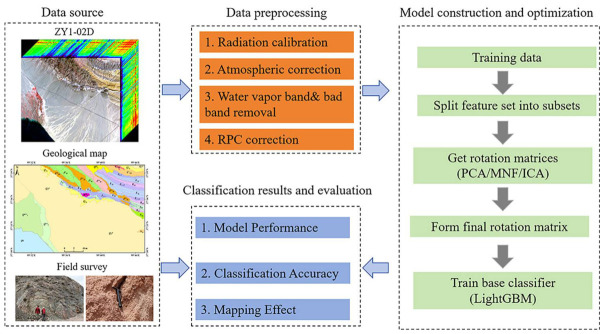
Flowchart of lithological mapping research.

This study adopts ZY1-02D satellite imagery as the data source to optimize the application performance of the ROF model for lithological classification. The optimized model is comprehensively evaluated from multiple perspectives, including model quantitative performance, classification accuracy, and mapping quality of lithological maps. The complete research flowchart is detailed in Figure 3.

### 3.1. Data Source and Processing

The ZY1-02D satellite was successfully launched on 12 September 2019 and is China’s first civilian hyperspectral operational satellite. It operates in a sun-synchronous orbit with a revisit period of 55 days and a designed lifespan of 8 years. Equipped with visible–near infrared and hyperspectral cameras, the satellite orbits at an altitude of 778 km, with a swath width of 60 km and a spatial resolution of 30 m. It provides a total of 166 spectral bands: 76 in the visible–near infrared range (395–1040 nm, spectral resolution of 10 nm) and 90 in the short-wave infrared range (1005–2501 nm, spectral resolution of 20 nm) (Table 1). This satellite is capable of conducting refined ground object spectral information surveys and can meet the needs of natural resource monitoring and investigation [45,46].

**Figure 4:**
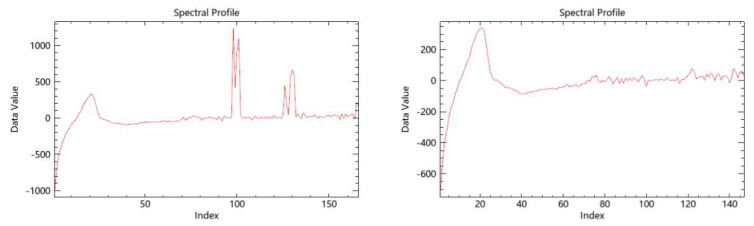
Comparison of water spectral curves before (**left**) and after (**right**) band removal.

Radiometric calibration was conducted on the image data using ENVI 5.6 software. Subsequently, atmospheric correction was performed on the images via the FLAASH module to obtain the real reflectance of ground objects. During the preprocessing of ZY1-02D data, it is necessary to eliminate overlapping spectral bands. Specifically, the spectral ranges of bands 72–76 and 77–79 overlap, and only bands 72–76 are retained. Meanwhile, water vapor absorption bands near 1.4 μm (bands 98–102) and 1.9 μm (bands 126–133), as well as severely noisy bands (bands 163–166) are removed to reduce interference on subsequent research. After the exclusion of these invalid bands, a total of 147 valid bands were finally retained for the lithological classification in this study (Figure 4).

### 3.2. ROF-LightGBM Model

This section elaborates the construction of the proposed ROF-LightGBM model. It first introduces the basic principle and workflow of rotation forest (ROF), then details three methods for rotation matrix construction, and finally describes LightGBM as the optimized base classifier.

#### 3.2.1. Rotation Forest

Rotation forest (ROF) is a homogeneous ensemble learning method that enhances base classifier diversity via feature rotation. It outperforms traditional ensemble methods (e.g., Bagging, AdaBoost) in many classification tasks by training diverse base classifiers on rotated feature subspaces [37,47].

Given a training set *T* = [*X*, *Y*], where $X=\left[x_{1},x_{2,}{,x}_{3},......,x_{n}\right]$, with *N* samples with *n* dimensional features; $Y=\left[y_{1},y_{2}{,y}_{3},......,y_{m}\right]$ and Y denotes the sample class labels, and each $y_{i}$ takes values from the set of class labels $y_{i}=\left\{l_{1},l_{2},l_{3},......,l_{c}\right\}$; $D=\left[D_{1},D_{2},D_{3},......,D_{L}\right]$, with L base classifiers; and *F* denote the feature subset.

Step 1 Feature partitioning: Randomly divide the feature subset *F* into *k* subsets $\Omega =\left\{\Omega_{1},\Omega_{2},...,\Omega_{k}\right\}$, where *k* is a preset parameter and each subset contains *r* features. The feature subsets $\Omega_{j}$ (1 ≤ *j* ≤ *k*) may be overlapping or non-overlapping; non-overlapping partitioning is generally adopted to enhance diversity. The number of features in each subset is *M* = *n*/*k*; if *n* is not divisible by *k*, the last subset will have fewer features.

Step 2 Bootstrap sampling: Let $F_{i,j}$ denote the *j*-th feature subset for training base classifier $D_{i}$. Extract columns corresponding to features in $F_{i,j}$ from matrix X to form a new matrix $X_{i,j}$. A 75% sample subset $X_{i,j}^{\prime }$ is drawn from $X_{i,j}$ via bootstrap sampling, which avoids identical coefficient matrix inputs to classifiers and ensures the diversity of the ensemble system.

Step 3 Rotation matrix generation: Perform PCA on $X_{i,j}^{\prime }$ to obtain $r_{j}$ principal component coefficients $a_{i,1}^{\left(0\right)},a_{i,1}^{\left(2\right)},\ldots {,a}_{i,1}^{\left(M_{j}\right)}$. During transformation of the PCA, some features may be zero, i.e., $M_{j}\leq M$. Subsequently, all principal component coefficient vectors are incorporated into a sparse rotation matrix.

(1) $$R_{i}=\left[\begin{array}{ccc} a_{i,1}^{\left(0\right)},a_{i,1}^{\left(2\right)},\ldots {,a}_{i,1}^{\left(M_{1}\right)} & \begin{array}{cc} \;\;\;\;[0] & \;\;\;\;\;\;\;\;\;\;\;\;\;\;\;\;\;\;\;\;\;\;\ldots \; \end{array} & \left[0\right]\\ \begin{array}{c} \left[0\right]\\ \vdots \end{array} & \;\;\begin{array}{c} a_{i,2}^{\left(0\right)},a_{i,2}^{\left(2\right)},\ldots {,a}_{i,2}^{\left(M_{2}\right)}\\ \vdots \end{array}\;\;\;\;\;\begin{array}{c} \ldots \\ \ddots \end{array}\;\;\;\;\;\;\; & \begin{array}{c} \left[0\right]\\ \vdots \end{array}\\ \left[0\right] & \;\;\;\begin{array}{cc} \left[0\right] & \;\;\;\;\;\;\;\;\;\;\;\;\;\;\;\;\;\;\;\;\ldots \end{array} & a_{i,K}^{\left(0\right)},a_{i,K1}^{\left(2\right)},\ldots {,a}_{i,K}^{\left(M_{K}\right)} \end{array}\right]$$

Step 4 Feature transformation: Reorder the column vectors of $R_{i}$ to obtain a new rotation matrix $R_{i}^{a}$, and train the classifier $D_{i}$ using the newly constructed training dataset ${XR}_{i}^{a}$.

Step 5 Base classifier training and Ensemble prediction: Transform each sample *x* by ${XR}_{i}^{a}$, generate *L* classification results via the base classifier $D_{i}$, and select the class $u_{w}(x)$ with the highest confidence as the category of *x*, where *C* denotes the total number of classes.

(2) $$u_{w}(x)=\frac{1}{L}\sum_{i=1}^{L}d_{i,j}(xA_{i}^{a}),w\in C$$

(3) $$x=argmax(u_{w}(x)),w\in C$$

#### 3.2.2. Rotation Matrix Construction

The diversity of ROF depends mainly on the rotation matrix. This section describes three widely used methods for constructing rotation matrices: PCA, MNF and ICA.

(1) PCA-based rotation matrix

For a D-dimensional random vector X($X\in R^{D}$), the goal is to find a projection direction *a* that maximizes the variance Φ(X) of the projection ($\alpha^{T}X$), which essentially involves solving the eigenvalues and eigenvectors of the covariance matrix [48]. In hyperspectral remote sensing images, PCA implementation includes three steps: calculating the band covariance matrix, performing eigenvalue decomposition to obtain eigenvalues *λ* and unit eigenvectors *α*, and deriving principal components via $Y_{i}=\alpha_{i}^{T}X$.

In ROF, these eigenvectors form the rotation matrix. For the j-th feature subset of the i-th base classifier, the covariance matrix of the bootstrap sample $X_{i,j}^{\prime }$ is computed as

(4) $$\sum =\mathrm{COV}(\;X_{i,j}^{\prime })$$

Eigende composition yields,

(5) $$\sum =U\wedge U^{T}$$

where U contains eigenvectors and Λ contains eigenvalues. The first $M_{j}$ eigenvectors are selected to construct the block $R_{i,j}$, which is assembled into the block-diagonal rotation matrix $R_{i}$.

(2) MNF-based rotation matrix

MNF is a noise-aware extension of PCA proposed by Green et al. [49], ordering components by signal-to-noise ratio (SNR) instead of variance. It involves two sequential PCA transformations: noise whitening followed by SNR-based component ordering.

Let be the total covariance matrix and $\sum_{N}$ the estimated noise covariance matrix. The generalized eigendecomposition is defined as

(6) $$\sum U=\lambda \sum_{N}U$$

where eigenvalues λ and eigenvectors u, form the transformation matrix U. The MNF-transformed components are obtained by $Y=U^{T}$, with components sorted in decreasing SNR.

In ROF, MNF eigenvectors are used for rotation matrix construction. For each bootstrap sample $X_{i,j}^{\prime }$, the noise-adjusted eigendecomposition is performed, and the first $M_{j}$ eigenvectors are taken as $R_{i,j}$. These blocks are combined into $R_{i}$, yielding noise-robust rotated features suitable for hyperspectral data.

(3) ICA-based rotation matrix

ICA is a blind signal separation technique used to estimate approximate values of mutually independent original signals from observed signals [50]. Assuming that the observed signal (X=A×S) is a linear combination of independent components *S*, both the mixing matrix *A* and the separation matrix *W* are unknown. Based on the assumptions that independent components *S* are statistically independent and non-Gaussian distributed, ICA estimates the mixing matrix *A*, then computes its inverse to obtain the separation matrix $W=A^{-1}$, thereby extracting the independent components (S=W×X).

In ROF, the ICA separation matrix *W* is directly employed as the rotation basis. For each bootstrap sample $X_{i,j}^{\prime }$, ICA is performed to obtain *W*, and the first $M_{j}$ rows of *W* are selected to form $R_{i,j}$. These blocks constitute the rotation matrix $R_{i}$, producing statistically independent rotated features and further enhancing ensemble diversity.

#### 3.2.3. LightGBM

LightGBM as base classifier is an efficient gradient boosting decision tree (GBDT) variant that maintains high accuracy while significantly accelerating training [51,52]. It runs over 20 times faster than conventional GBDT on public datasets with comparable performance [31], making it suitable for high-dimensional remote sensing data and to be adopted as the base classifier in ROF.

(1) Gradient-based One-Side Sampling (GOSS):

Retain the top a × 100% large-gradient instances as set A, randomly extract a subset B with size $b\times \left|A^{c}\right|$ from the remaining small-gradient instances set $A^{c}$; complete data splitting on the merged subset A∪B based on the estimated variance gain $\tilde{V_{j}}(d)$.

(2) Exclusive Feature Bundling (EFB):

Bundle mutually exclusive sparse features into a single feature bundle. The complexity of histogram construction reduces from “data volume × number of features” to “data volume × number of feature bundles”, significantly accelerating training.

(3) Histogram-Based Decision Tree

Discretize continuous features into bins and build histograms. It reduces memory usage and speeds up split finding via histogram difference.

(4) Leaf-wise Growth Strategy with Depth Constraint

**Figure 5:**
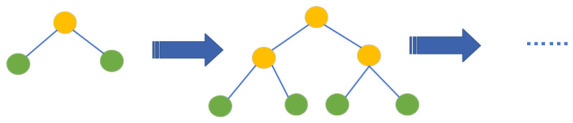
Level-wise growth strategy.

**Figure 6:**
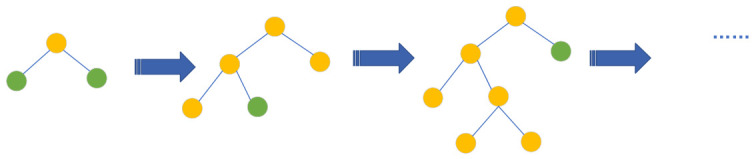
Leaf-wise growth strategy.

Replaces the traditional Level-wise growth strategy (Figure 5 and Figure 6). Grow the leaf with maximum gain each time, instead of level-wise splitting. A maximum depth is set to avoid overfitting.

### 3.3. Random Forest

Random forest (RF) is a Bagging-based ensemble classifier with CART trees as base learners, introducing randomness in both data sampling and feature selection to enhance generalization [18]. It generates L training subsets via bootstrap sampling, each containing approximately two-thirds of the original data, with the remaining one-third used as out-of-bag (OOB) data for error estimation. Let F be the total number of features. At each node split, f features (f (f < F)) are randomly selected, and the optimal split is determined by the Gini coefficient. CART trees are built through recursive splitting until stopping criteria are satisfied, eliminating the need for pruning due to randomization. The final classification result is obtained via majority voting among all trees [52,53].

In classification tasks, the Gini coefficient measures the impurity of class distribution. Let C denote the total number of classes, and $p_{k}$ the probability that a sample belongs to the k-th class. The Gini coefficient is defined as:

(7) $$Gini(p)=\sum_{k=1}^{K}p_{k}(1-p_{k})\;=1-\sum_{k=1}^{K}p_{k}^{2}$$

Given a sample set D, the Gini coefficient is calculated as follows:

(8) $$Gini(p)=1-{(1-\sum_{k=1}^{K}\frac{\left|C_{k}\right|}{\left|D\right|})}^{2}$$

where $C_{k}$ denotes the subset of samples belonging to the k-th class in D, and k represents the number of classification categories.

This study was implemented based on Python 3.8. All experiments were conducted on a computer equipped with an Intel Core i9-12900H CPU (2.50 GHz) and 16 GB RAM. Hyperspectral data preprocessing was completed in ENVI 5.6, and spatial mapping results were finalized using ArcGIS 10.5.

## 4. Results

### 4.1. Sample Data Selection

**Figure 7:**
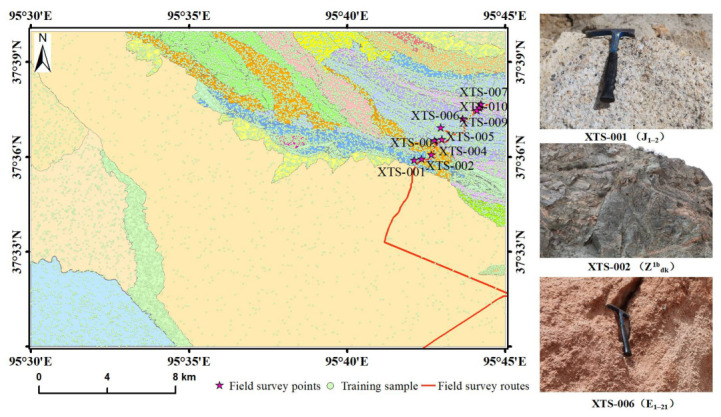
Distribution map of field verification points and sample points in the study area.

In the Xitieshan study area, due to the good outcrop condition of strata, the verification of the boundary points of different stratigraphic lithological units was conducted along the gullies. The field survey routes, verification points, and on-site photos of typical stratigraphic lithology are detailed in Figure 7.

The quality of training sample dataset selection is a critical factor determining the performance of subsequent machine learning classification. In this study, training samples were selected based on the geological map of the study area and field survey results. A random sampling strategy was adopted to select samples from different rock units, and samples at the boundaries of different stratigraphic rock units were excluded to avoid the impact of mislabeling on lithological classification (Table 2).

### 4.2. Optimum Model Parameter Settings of the ROF-LightGBM Model

Key parameters affecting ROF model performance include the number of LightGBM base classifiers (L), trees in the model (T), and features per subset (M). Referring to relevant studies [37], M = 2 when total features < 10 and M = 3 when ≥ 10. Since ZY1-02D data has over 10 bands, M = 3.

In the classification research, the number of trees for RF and LightGBM models was set to T = 100 by default. To compare lithological mapping results of the ROF-LightGBM model with RF and LightGBM under the same conditions, the total number of trees in the ROF-LightGBM model was also set to 100. Since the total number of trees in this model is L × T, different L × T combinations (50 × 2, 25 × 4, 10 × 10, 4 × 25, 2 × 50) were set under the premise of keeping bootstrap unchanged [39]. After 10 cycles for each combination, the optimal L × T combination was comprehensively determined based on accuracy and running time.

As shown in Table 3, comparison of the ROF-LightGBM model under different L × T combinations indicates the 50 × 2 combination achieved the lowest average classification accuracy (71.98%) but the smallest standard deviation, showing strong stability; the 4 × 25 combination yielded the optimal accuracy (75.29%). Significance analysis at the 0.05 level revealed that the 50 × 2 and 25 × 4 combinations exhibited significantly inferior classification performance compared to 10 × 10, the 4 × 25 combinations, which demonstrated significantly superior performance, and no significant difference was observed between the 2 × 50 and 10 × 10 combinations.

Comparison of the ROF-LightGBM model running time across different L × T combinations showed that L significantly affected the training time, which decreased with the reduction in the number of classifiers. The optimal 4 × 25 combination exhibited a training time of 11.41 s (nearly 10-fold shorter than that of the 50 × 2 combination), with marginally improved classification accuracy.

In summary, the ROF-LightGBM model achieved optimal classification performance, stability, and efficiency under the L × T combination of 4 × 25.

With L = 5 and T = 25, bootstrap sampling ratios of 25%, 50%, and 75% were tested to compare model performance in study area (Table 4). Different bootstrap ratios had minimal impact on model performance, with no significant differences in accuracy or running time observed. The 75% ratio yielded the highest accuracy but longer training time, while the 25% ratio showed the lowest accuracy but shorter training time. Significance tests revealed that the 75% sampling method was significantly superior to 50% and 25%. Thus, the 75% bootstrap sampling ratio was adopted for subsequent research.

After setting the parameters such as L, T and bootstrap, the parameter boundaries for the other three hyperparameters, namely learning_rate, max_depth, and reg_alpha, were defined as (0.001, 0.9), (3, 12), and (0.01, 0.9), respectively. Multiple groups of parameter combinations were randomly generated. Additionally, during the training process, a ten-fold cross-validation procedure was implemented to evaluate the overall performance of the proposed ROF-LightGBM model. By comparing the results with those obtained using the default parameter settings, it was finally determined that the model with learning_rate = 0.1, max_depth = −1, and reg_alpha = 0 achieved the best classification performance.

### 4.3. Lithological Mapping Results of Different Machine Learning Models

An evaluation of lithological classification performance between the ROF-LightGBM model (L = 4, T = 25) and the RF and LightGBM models (T = 100) showed that with the same number of decision trees, the LightGBM model completed training in the shortest time, totaling 3.82 s. It achieved better classification accuracy while reducing training time by nearly half compared to the RF model. The ROF-LightGBM model performed best, with its average classification accuracy and Kappa coefficient being 2.48% and 0.02 higher than those of the LightGBM model, respectively (Table 5).

**Figure 8:**
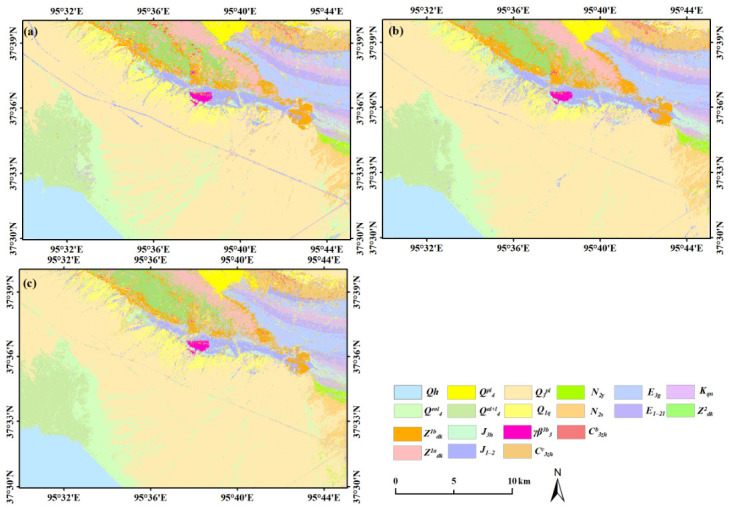
Lithological mapping results of different machine learning methods. (**a**) RF; (**b**) LightGBM; and (**c**) ROF.

It can be concluded that the performance of the LightGBM classifier was superior to that of traditional machine learning models (e.g., decision trees, random forests) in this lithological classification research. Employing LightGBM as the base classifier significantly enhanced the performance of the ROF model. Figure 8 shows that the classification results obtained by LightGBM yield the best integrity of rock units with the least noise; those obtained by ROF are almost not affected by linear ground features, but the integrity of rock units is the worst.

**Figure 9:**
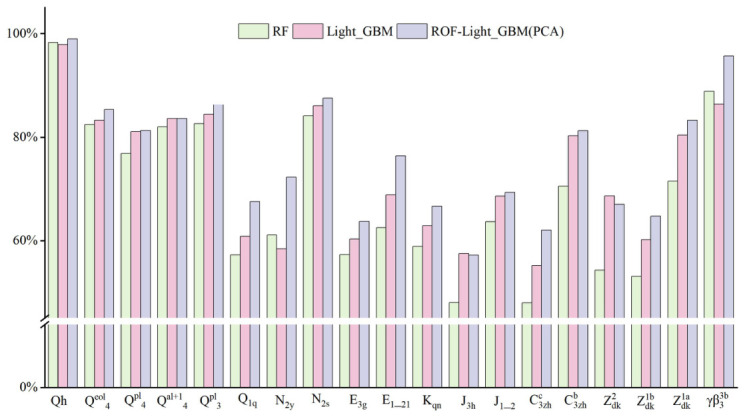
Rock units classification accuracy of different classification methods in the study area.

Under the condition of the same number of classifiers (L = 4), the overall classification accuracy and kappa coefficient of ROF-LightGBM were increased by 8.32% and 0.09, respectively, compared with ROF, and the model training time was shortened by 31.53%. As shown in Figure 9, the classification accuracy of ROF-LightGBM for all rock units was superior to that of ROF. The classification accuracy of 12 rock units was improved by nearly or more than 10%, among which $Q_{1q}$, $N_{2y}$ and $C_{3zh}^{C}$ exhibited the largest increases in classification accuracy, with their average classification accuracies increased by 15.00%, 22.23%, and 19.53%, respectively.

A further comparative analysis of lithological classification performance between ROF and ROF-LightGBM under T = 100 revealed that the traditional ROF model required constructing 100 base classifiers to generate 100 decision trees, significantly increasing the time cost of model training. With the same number of decision trees, the ROF model consumed nearly 43 times more training time than ROF-LightGBM, while the overall classification accuracy was only improved by 1.19%. When the number of base classifiers in ROF-LightGBM was increased to 100, the classification accuracy decreased by 2.2% compared with the ROF model (L = 100), whereas the classification time was only one-fourth that of the ROF model. Compared with ROF-LightGBM (L = 4), the classification accuracy decreased by 1.01%, but the classification time increased by nearly 10 times. These results indicate that ROF-LightGBM can rapidly achieve superior classification performance through flexible parameter combinations.

**Figure 10:**
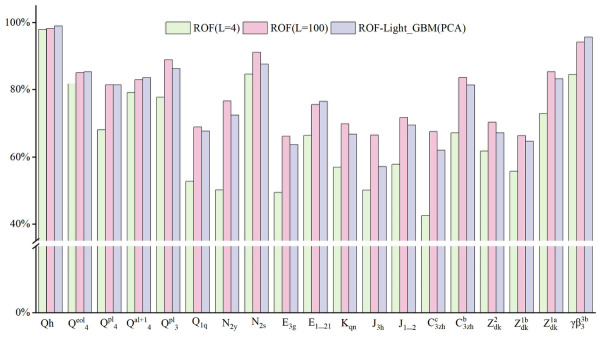
Rock units classification accuracy of ROF and its variation model methods in the study area.

Among different stratigraphic lithological units, $N_{2y}$, $J_{3h}$ and $C_{3zh}^{C}$ showed substantial accuracy improvements of 4.20%, 9.29% and 5.49%, respectively, while Qh, $Q_{4}^{eol}$, $Q_{4}^{al+1}$, $E_{1-21}$ and ${\gamma \beta }_{3}^{3b}$ showed various degrees of accuracy reduction of 0.70%, 0.28%, 0.65%, 0.9% and 1.53%, respectively (Figure 10).

### 4.4. Lithological Mapping Results of ROF-LightGBM with Different Rotation Matrix Constructors

In addition to PCA, MNF and ICA are also commonly used feature extraction methods in lithological classification studies. Therefore, MNF and ICA were used for rotation matrix construction to identify the most favorable approach for lithological classification in this study.

Comparison of different rotation matrix construction methods (Table 6) indicated that the ROF-LightGBM model with ICA-derived rotation matrix exhibited a 4-fold increase in running time compared to that with PCA. In contrast, the MNF-based model showed only a 0.64% increase in running time but a 7.89% improvement in classification accuracy, representing the most significant performance enhancement, with an overall classification accuracy of 82.17% and a kappa coefficient of 0.81.

**Figure 11:**
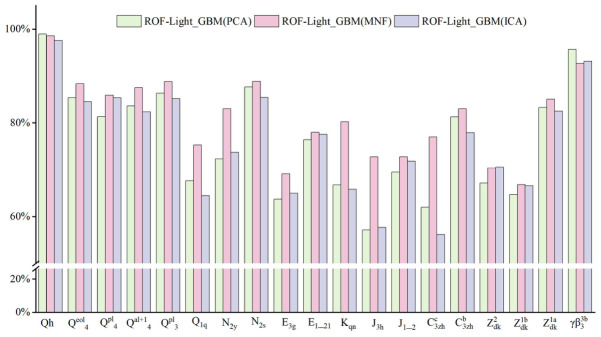
Rock units classification accuracy of ROF-LightGBM under different rotation matrix models.

Figure 11 presents the classification accuracy of different lithological units using three distinct rotation matrices in the study area. The results indicate that, except for the ${\gamma \beta }_{3}^{3b}$, the ROF-LightGBM model with PCA-derived rotation matrix achieved optimal accuracy; all other units exhibited the highest classification accuracy with the MNF-derived rotation matrix. Notably, the recognition accuracies of $N_{2y}$, $K_{qn}$, $J_{3h}$ and $C_{3zh}^{C}$ were significantly superior to those of the other two models.

**Figure 12:**
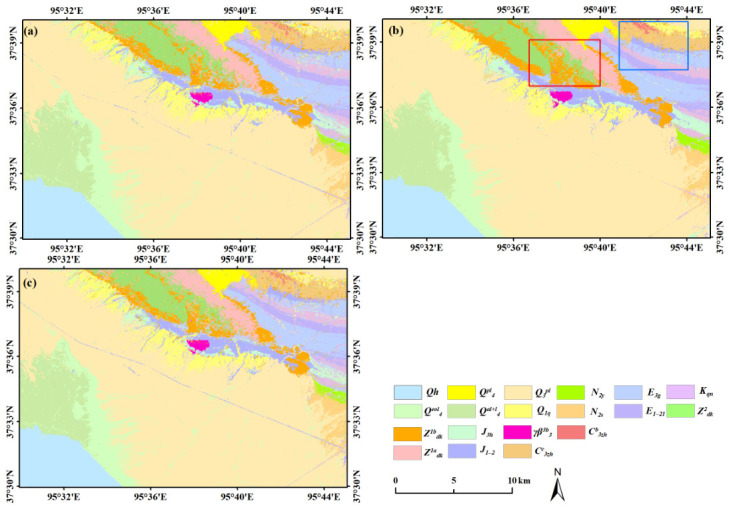
Lithological mapping results of the RoF-LightGBM model with different rotation matrix constructors in the Xitieshan study area. (**a**) ROF-LightGBM (PCA); (**b**) ROF-LightGBM (MNF); and (**c**) ROF-LightGBM (ICA).

Figure 12 showed that ROF-LightGBM, compared to LightGBM, significantly reduced fine fragmented patches, improved the completeness and clarity of stratigraphic boundaries, and mitigated the influence of linear features, with ROF-LightGBM (MNF) demonstrating the most prominent effects. Moreover, ROF-LightGBM (MNF) achieved clearer boundary delineation for $Z_{dk}^{1a}$, $Z_{dk}^{1b}$ and $Z_{dk}^{2}$ units than its counterparts with PCA- or ICA-derived rotation matrices and yielded significantly superior areal integrity for $C_{3zh}^{b}$ unit.

### 4.5. Impact of Number and Quality of Training Samples on ROF-LightGBM

Sample quantity is a key factor affecting the accuracy of lithology classification models. When the proportion of sample quantity decreases from 100% to 25%, the accuracy of all classification models shows a continuous downward trend, and the ROF-LightGBM integrated model has significantly better adaptability to the reduction of sample quantity than the single models of ROF, LightGBM, and RF. As shown in Table 7, the ROF-LightGBM integrated model has the smallest accuracy loss, with a 7.3% accuracy decrease under the condition of 25% sample quantity; ROF and RF are more sensitive to changes in sample quantity, with accuracy reduction ranging from 9.21% to 9.60%, and the accuracy reduction of the LightGBM model is 8.70%, both higher than that of the ROF-LightGBM integrated model.

The classification performance of the ROF-LightGBM model varies significantly with different rotation matrix construction methods and parameter settings (number of base classifiers L, number of decision trees T) when the sample quantity changes. Notably, parameter optimization is more effective in reducing the impact caused by sample quantity reduction than adjusting the rotation matrix construction method. Specifically, regarding rotation matrix construction: the ROF-LightGBM (MNF) model achieves the highest initial accuracy but is highly sensitive to changes in sample quantity, with an accuracy reduction of 9.92% when the sample quantity decreases to 25%. In contrast, increasing L and T can enhance the model’s adaptability to reduced sample quantity; when L = 4 and L = 100, the model’s accuracy reduction is 6.15% and 6.45%, respectively, after the sample quantity decreases to 25%.

As shown in Table 8, when the proportion of mislabeled samples increases from 0% to 45%, the classification accuracy of all models shows a significant downward trend. Among them, ROF (PCA, L = 100) and ROF-LightGBM (PCA, L = 100) experience the least impact from mislabeled samples. When the proportion of mislabeled samples reaches 45%, the accuracy reduction of the two models is 7.61% and 4.42%, respectively. This indicates that adjusting the number of base classifiers (L) of the ROF model can effectively mitigate the negative impact of mislabeled samples on the lithology classification accuracy in the Xitieshan area.

Different rotation matrix construction methods also affect the anti-interference ability of the ROF-LightGBM model against mislabeled samples: the model with PCA-constructed rotation matrix exhibits lower susceptibility to mislabeled samples than those using MNF and ICA. Specifically, despite the highest initial accuracy (82.17%) of the ROF-LightGBM (MNF) model, its accuracy reduction reaches 12.14% (final accuracy: 70.03%) when the proportion of mislabeled samples reaches 45%, indicating the poorest anti-interference stability.

In conclusion, both sample quantity and quality have a significant influence on lithology classification accuracy in the Xitieshan area. The ROF-LightGBM integrated model outperforms the single models of ROF, LightGBM, and RF in terms of both adaptability and anti-interference ability. Additionally, the ROF-LightGBM model constructed with MNF rotation matrix achieves the optimal classification accuracy but exhibits high sensitivity to changes in sample quantity and quality. Fortunately, optimizing the parameters of the ROF-LightGBM model can effectively mitigate the adverse impact of such changes on its classification accuracy.

## 5. Discussion

**Figure 13:**
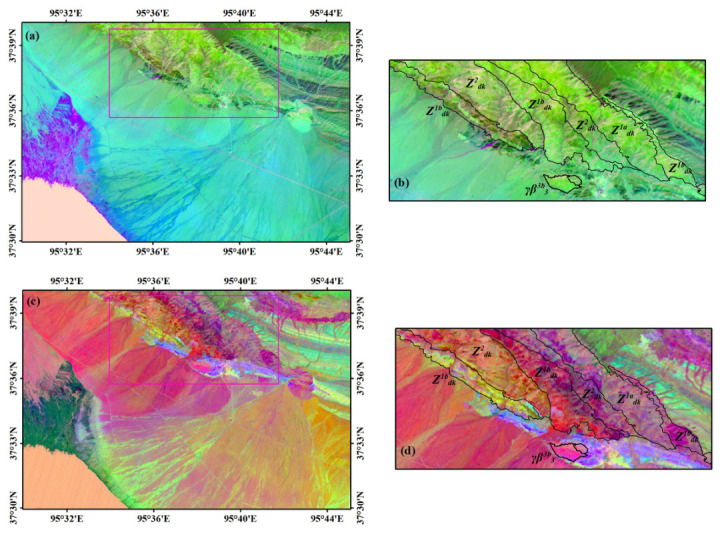
Image enhancement of the study area. (**a**) PCA (R: Component 1; G: Component 2; B: Component 3); (**b**) magnified view of (**a**); (**c**) MNF (R: Component 2; G: Component 3; B: Component 4); and (**d**) magnified view of (**c**).

In this study, the proposed ROF-LightGBM (MNF) model effectively enhanced the accuracy and efficiency of lithological classification in the Xitieshan study area, providing a reliable technical support for lithological mapping. In contrast to PCA, which constructs rotation matrices by maximizing global variance, the MNF transform ranks spectral components based on signal-to-noise ratio (SNR), emphasizing high-SNR features and suppressing noise interference. This makes MNF well-suited for hyperspectral lithological mapping [42,54]. Rotation forest improves classification performance by randomly partitioning features and performing independent rotations to increase base classifier diversity. The SNR-guided property of MNF provides optimized, noise-robust rotation directions for each subset, maintaining subset independence while reducing noise during rotation. Furthermore, the MNF transformation applied to the shortwave infrared bands of ZY1-20D enables better discrimination of metamorphic strata such as $Z_{dk}^{1a}$, $Z_{dk}^{1b}$ and $Z_{dk}^{2}$, as well as the ${\gamma \beta }_{3}^{3b}$ intrusive rock formation (Figure 13). Therefore, the ROF-LightGBM model employing MNF for rotation matrix construction achieves superior classification performance in this study.

**Figure 14:**
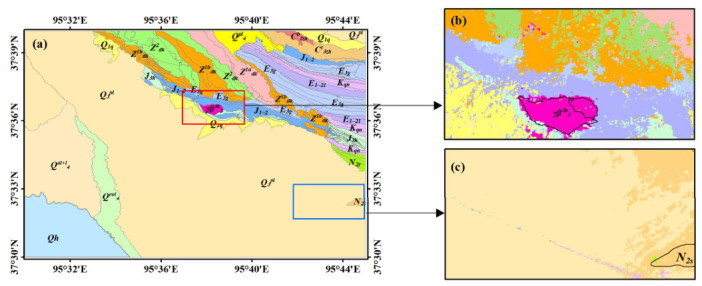
Local comparison map of lithological classification. (**a**) Geological map of the Xitieshan study area; (**b**) lithological mapping results of ROF-LightGBM (MNF) (red framed area); and (**c**) lithological mapping results of ROF-LightGBM (MNF) (blue framed area).

As one of the important influencing factors in lithological classification model, training samples exert a crucial impact on the final classification results. In addition to the quantity and quality of samples, the imbalance of samples among different lithological units also affects the classification results to varying degrees. Although model optimization can improve the classification accuracy of lithological units to a certain extent, the final classification results are still prone to misclassification into lithological units with similar lithological characteristics. A typical example is the $N_{2S}$ rock unit, mainly composed of massive grayish-green and yellowish-gray mudstone with a small outcrop area (0.12% of the study area) and extremely limited training samples. Due to its similar spectral and lithological characteristics to $Q_{3}^{pl}$, the classification results show obvious mixed classification and unclear boundaries (Figure 14c). In contrast, the intrusive rock unit ${\gamma \beta }_{3}^{3b}$ (purple biotite granite) has distinct lithological and spectral differences from surrounding $J_{2-1}$ and $Q_{1q}$ (Figure 14b). Even with limited samples, it can be clearly distinguished. In summary, sample imbalance causes model bias, reducing minority unit classification accuracy. It significantly affects units with small areas, limited samples and similar features, but its impact is alleviated for units with distinct characteristics, requiring models to enhance robustness to sample imbalance.

In existing research, scholars have adopted various optimization strategies to improve the performance of the ROF model and successively proposed a series of optimized models such as RRF [37], ROF-GBM [39], and RotBoost [55]. These optimized methods have indeed improved the classification accuracy of the original ROF model to a certain extent, effectively addressing the limitations of the traditional ROF model in feature extraction and classification generalization. However, a prominent problem of these optimization approaches is the high time cost, which is particularly obvious in the classification task of high-dimensional data (e.g., hyperspectral remote sensing data for lithological classification), severely restricting their practical application in large-scale and high-efficiency research scenarios. Taking the lithological classification of the study area as an example, the ROF-GBM model takes a total of 694.94 s to complete the classification task, with a classification accuracy and Kappa coefficient of 73.72% and 0.72, respectively. Although its classification results are slightly superior to those of the traditional random forest (RF) and LightGBM models, the classification time is significantly increased; it is even dozens of times longer than that of the LightGBM model, which greatly reduces the efficiency of lithological classification research. In sharp contrast, the proposed ROF-LightGBM (MNF) model achieves a significant balance between classification accuracy and efficiency. The training time of this model is only 4.72 s, which is far shorter than that of the ROF-GBM model, while its classification accuracy and Kappa coefficient reach 82.17% and 0.81, respectively. This indicates that the ROF-LightGBM (MNF) model not only effectively reduces the time cost of model training and classification but also significantly improves the classification accuracy and reliability. Compared with the existing ROF-based optimized models, it has more obvious advantages in practical lithological classification research, providing a more efficient and accurate technical approach for large-scale lithological mapping and related geological research.

**Figure 15:**
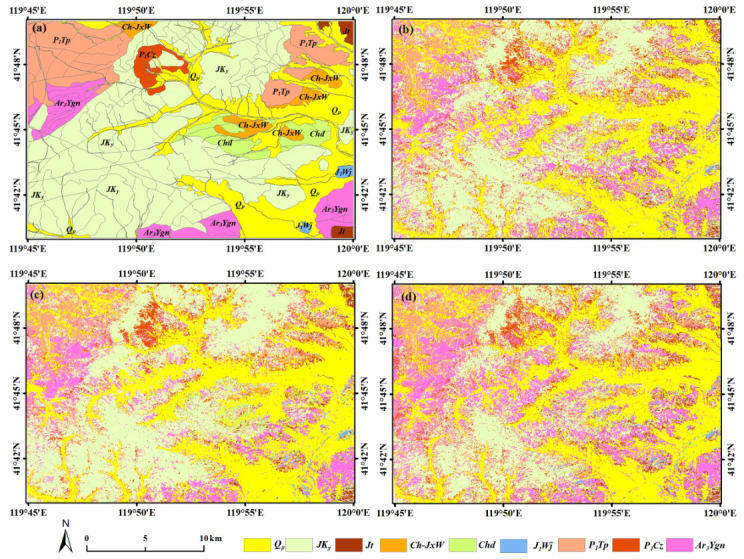
Lithological mapping results of the RoF-LightGBM model with different rotation matrix constructors in a vegetation-covered study area. (**a**) Lithological map; (**b**) ROF-LightGBM (PCA); (**c**) ROF-LightGBM (MNF); and (**d**) ROF-LightGBM (ICA).

Due to the weak signals and interference from vegetation cover, remote sensing-based detection of rock information in vegetated areas poses challenges. To explore the applicability of the ROF-LightGBM model in vegetation-covered areas, this study further verified its classification performance. Compared with the ROF-LightGBM model with a rotation matrix constructed by PCA (Table 9), the one using MNF for rotation matrix construction achieved a 5.22% improvement in overall classification accuracy, with only a 3.03% increase in training time. In contrast, the model using ICA for rotation matrix construction exhibited a significant increase in time consumption (approximately 7-fold higher) and a 1.2% decrease in classification accuracy compared with the PCA-based ROF-LightGBM model, which is consistent with its performance in the Xitieshan bedrock-exposed areas. However, the lithological classification accuracy in vegetation-covered area is significantly lower than that in the Xitieshan bedrock-exposed areas, with more noise in the classification results and indistinct boundaries of some rock units (Figure 15).

To further critically examine the scenarios causing reduced model performance, dense vegetation coverage emerges as the primary limiting factor. Even low vegetation coverage (~10%) significantly weakens or masks bedrock spectral signals, while dense vegetation introduces strong spectral interference that obscures subtle lithological differences. Furthermore, topographic complexity and terrain shadow act as additional confounding factors, as undulating terrain in vegetated areas causes inconsistent illumination and shadows that distort spectral signatures and add classification noise. Moreover, shallowly weathered rocks show limited spectral separability: partial exposure and thin weathered layers make their spectra similar to surrounding soil and vegetation, weakening the model’s ability to reliably distinguish lithological units [56,57,58].

**Figure 16:**
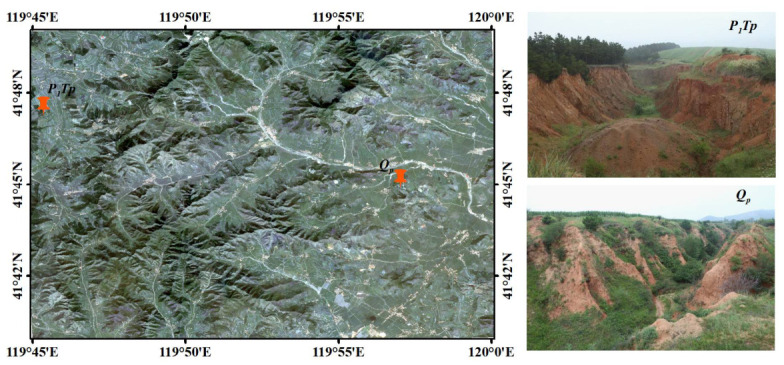
Field verification points and photos of vegetation-covered areas.

In this study, the $P_{1}TP$ and $Q_{p}$ units are mainly covered by corn crops with similar morphology and spectra, resulting in severe spectral mixing and inter-class confusion that the ROF-LightGBM model struggles to resolve. Additionally, intense weathering and thick overlying soil exacerbate performance degradation. The $P_{1}TP$ unit has a weathering layer over 10 m, with continuous soil and vegetation decoupling surface reflectance from bedrock properties, increasing classification noise and blurring lithological boundaries, with effects far more severe than in bedrock-exposed areas (Figure 16).

This indicates that model optimization can moderately improve the lithological classification accuracy and efficiency in vegetation-covered areas. Nevertheless, relying on single-source data and traditional data mining approaches is insufficient to meet the requirements of lithological classification in vegetation-covered regions. Therefore, future research will further investigate the remote sensing mechanism of rocks and minerals in vegetation-covered study areas, as well as the lithological classification based on multi-source data fusion.

The number of base classifiers (L), decision trees (T) and features in each subset (M) are the main parameters affecting the classification performance of the ROF-LightGBM (MNF) model. A control variable method was adopted to investigate the effects of individual parameter variations on model performance, with the results presented in Figure 15.

(1) Effect of base classifier number (L) on model performance

In the Xitieshan study area, the classification accuracy exhibited the most significant variation when L increased from 1 to 4, with an increase of 3.73%. Subsequently, the accuracy increased slowly with the increase of L, reaching the maximum value of 83.40% when L = 40. Compared with L = 4, the accuracy was improved by 1.23%, but the time consumption increased by approximately 10 times, totaling 46.76 s.

**Figure 17:**
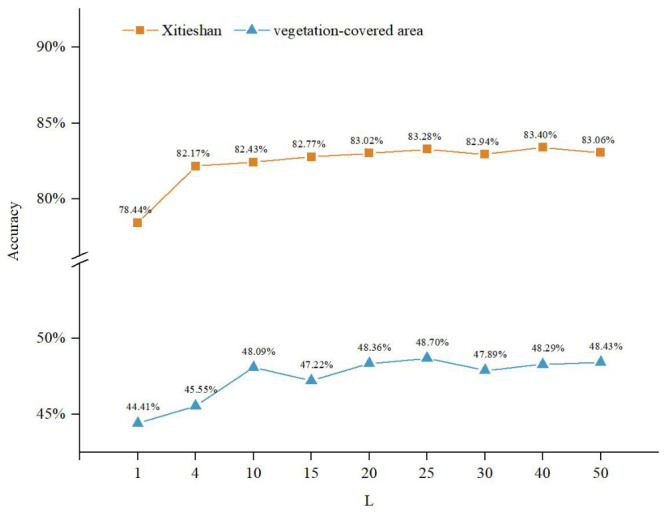
Lithological mapping accuracy of ROF-LightGBM (MNF) under different numbers of base classifiers (L).

In the vegetation-covered study area, the classification accuracy changed most drastically when L increased from 1 to 10, with an increase of 3.68%. Afterwards, the accuracy fluctuated slightly with the increase of L, achieving the optimal value of 48.70% when L = 25. Compared with L = 10, the accuracy was only improved by 0.60%, while the model training time increased by more than 3 times, totaling 14 s (Figure 17).

(2) Effect of decision tree number (T) on model performance

**Figure 18:**
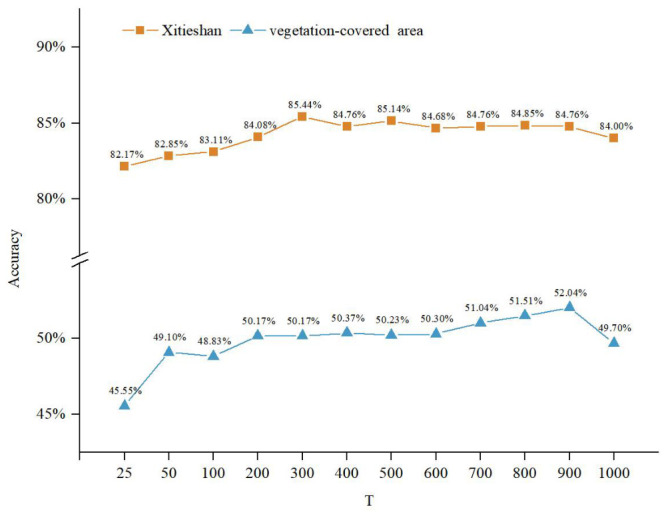
Lithological mapping accuracy of ROF-LightGBM (MNF) under different numbers of decision trees (T).

In the Xitieshan study area, the classification accuracy increased gradually as T increased from 25 to 400, reaching the highest value of 85.44% when T = 300 (time consumption: 33.14 s). Compared with T = 25, the accuracy was improved by 3.27%, and the time consumption increased by approximately 8 times. When T exceeded 400, the classification accuracy decreased to varying degrees (Figure 18).

In the vegetation-covered area, the classification accuracy increased most significantly when T increased from 25 to 50, with a total increase of 3.55% and a time increase of 1.78 s. Subsequently, the accuracy increased slowly with the increase of T, reaching the maximum value of 52.04% when T = 900. Compared with T = 25, the time consumption increased by approximately 22 times. When T exceeded 900, the classification accuracy decreased significantly.

(3) Effect of feature number in subset (M) on model performance

**Figure 19:**
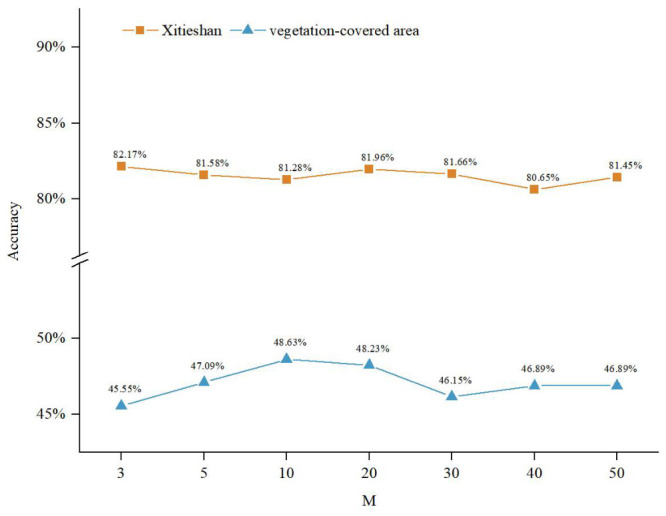
Lithological mapping accuracy of ROF-LightGBM (MNF) under different number feature numbers (M) in subset.

In the Xitieshan study area, the classification accuracy decreased to varying degrees with the increase of M in the feature subset. In the vegetation-covered study area, the classification accuracy reached the highest value of 48.63% when M increased to 10; when M exceeded 10, the accuracy decreased with the increase of M. During the variation of M, there was no significant change in the model training time (Figure 19).

After combining the optimal solutions of each parameter, the model classification accuracy was 84.16% with a total time consumption of 403 s when L = 40, T = 400, and M = 3 in the Xitieshan study area, showing a slight decrease in accuracy compared with the single-parameter T optimal value. In the vegetation-covered area, the model classification accuracy was improved to 57.19% with a total time consumption of 527.04 s when L = 50, T = 900, and M = 10. It can be deduced that the assembly of individually optimized parameters is not conducive to the attainment of a globally optimal model. Instead, a holistic and systematic parameter calibration framework should be established to strike a delicate trade-off between classification accuracy and computational efficiency, with a view to optimizing the overall performance of the model in practical lithological classification research.

Collectively, these findings highlight the inherent limitations of the model. Dense vegetation cover is the core bottleneck restricting model performance. While it does not completely negate the applicability of the ROF-LightGBM framework, the model can only be reliably applied in areas with moderate vegetation coverage, thin weathering layers, and distinct lithological spectral signatures. Once vegetation coverage increases, model accuracy declines sharply, reflecting its poor generalization capability. Future research should prioritize integrating multi-source data such as radar and thermal infrared data to effectively mitigate vegetation interference and overcome the model’s application limitations.

## 6. Conclusions

To improve the accuracy and efficiency of lithological classification with high-dimensional remote sensing data, this study focuses on optimizing the rotation forest (ROF) framework for lithological mapping. The key optimizations include replacing the base classifier from traditional decision trees to LightGBM and introducing MNF to construct the rotation matrix. Through these synergistic improvements, the ROF-LightGBM model based on the MNF-constructed rotation matrix was established.

Experimental results demonstrate that the ROF-LightGBM model achieves significantly higher lithological classification accuracy with shorter training time, clearly outperforming both the conventional ROF, RF and LightGBM models. The optimized model also exhibits stronger robustness against data disturbances, maintaining more stable classification performance as training samples decrease and erroneous or noisy samples increase. In addition, the approach used to construct the rotation matrix plays a critical role in model performance. The MNF-based method shows distinct advantages in lithological mapping accuracy and yields a favorable balance between classification precision and computational efficiency.

Nevertheless, the model still exhibits certain limitations in practical applications. Specifically, it achieves relatively poor classification performance for stratigraphic units with small outcrop areas, which may be caused by limited sample representativeness and weak spectral separability in narrow outcrop zones, and it also fails to yield satisfactory results in densely vegetation-covered areas due to the strong interference and masking effects of vegetation on the spectral signals of underlying bedrock, which restrict accurate lithological identification.

Further research will concentrate on integrating multi-source remote sensing data to improve the accuracy of lithological classification, especially in vegetation-covered areas. Empirical validation in typical geological regions will be carried out to strengthen model applicability, so as to provide theoretical and technical support for the large-scale application of remote-sensing-based lithological identification.

## Figures and Tables

**Table 1:** Main specifications of the ZY1-02D HSI.

Specifications	ZY1-02D HIS	
Wavelength (μm)	0.4–0.25	
Spectral bands	VINR:76	SWIR:90
Spectral resolution (nm)	10	20
Swath width (m)	30	
Spatial resolution (km)	60	
Slew capability	±26°	
Revisit	3 days	
Global coverage	55 days	

**Table 2:** Statistical table of sample distribution in the study area.

Index	Rock Type	Area (km^2^)	Number of Samples	Index	Rock Type	Area (km^2^)	Number of Samples
1	Qh	16.28	642	11	K_qn_	7.71	765
2	Q^eol^_4_	11.03	704	12	J_3h_	6.04	604
3	Q^pl^_4_	4.71	381	13	J_1–2_	10.14	1015
4	Q^al+l^_4_	37.38	416	14	C^c^_3zh_	0.96	196
5	Q_3_^pl^	226.26	949	15	C^b^_3zh_	6.67	803
6	Q_1q_	9.97	458	16	Z^2^_dk_	14.07	737
7	N_2y_	1.87	219	17	Z^1b^_dk_	16.47	922
8	N_2S_	0.52	200	18	Z^1a^_dk_	8.97	798
9	E_3g_	20.18	989	19	γβ^3b^_3_	0.92	332
10	E_1–21_	6.62	650				

**Table 3:** Mean values and standard deviations of classification accuracies and running times of the RoF-LightGBM model under different L and T combinations.

L × T	OA	Time
50 × 2	71.98% ± 0.12% ○	94.28 s
25 × 4	73.13% ± 0.36% ○	49.55 s
10 × 10	74.60% ± 0.52%	21.98 s
4 × 25	75.29% ± 0.79% ★	11.41 s
2 × 50	74.27% ± 0.49%	7.69 s

★ 10 × 10 is significantly better; ○ 10 × 10 is significantly worse, at the significance level = 0.05.

**Table 4:** Mean values and standard deviations of classification accuracies and running times of the RoF-LightGBM model under different bootstrap values.

Bootstrap	0A	Time
75%	75.25% ± 0.60%	4.85
50%	74.92% ± 0.68% ○	4.28
25%	73.43% ± 0.34% ○	3.20

○ 25% and 50% are significantly worse, at the significance level = 0.05.

**Table 5:** Classification accuracy and running time of different machine learning models.

Model	OA	Kappa	Time
RF	67.74%	0.66	6.87 s
LightGBM	72.75%	0.70	3.82 s
ROF (PCA; L = 4)	65.96%	0.64	6.85 s
ROF (PCA; L = 100)	75.47%	0.74	172.16 s
ROF-LightGBM (PCA; L = 4)	74.28%	0.74	4.69 s
ROF-LightGBM (PCA; L = 100)	73.27%	0.72	46.17 s

**Table 6:** Classification accuracy and running time of the RoF-LightGBM model under different rotation matrix constructors in the Xitieshan area.

Model	OA	Kappa	Time
ROF-LightGBM (PCA)	74.28%	0.73	4.69 s
ROF-LightGBM (MNF)	82.17%	0.81	4.72 s
ROF-LightGBM (ICA)	75.42%	0.74	17.23 s

**Table 7:** Classification accuracy of machine learning models with different numbers of training samples.

Models	Number of Training Samples (%)				Accuracy Reduction
	100%	75%	50%	25%	
ROF (PCA, L = 4)	67.61%	66.25%	63.07%	58.40%	9.21%
ROF (PCA, L = 100)	75.47%	75.21%	69.95%	65.87%	9.60%
LightGBM	72.75%	71.69%	67.82%	64.05%	8.70%
RF	67.74%	65.79%	62.78%	58.15%	9.59%
ROF-LightGBM (PCA)	74.28%	73.30%	72.45%	66.98%	7.30%
ROF-LightGBM (PCA, L = 4)	77.54%	76.74%	75.17%	71.39%	6.15%
ROF-LightGBM (PCA, L = 100)	76.78%	75.93%	73.85%	70.33%	6.45%
ROF-LightGBM (MNF)	82.17%	80.65%	78.44%	75.25%	9.92%
ROF-LightGBM (ICA)	75.42%	73.30%	67.78%	68.33%	7.09%

**Table 8:** Classification accuracy of machine learning models with different numbers of mislabeling training samples.

Models	Mislabeling Training Samples				Accuracy Reduction
	0%	15%	30%	45%	
ROF (PCA, L = 4)	67.61%	59.85%	50.52%	42.44%	25.17%
ROF (PCA, L = 100)	75.47%	73.60%	72.11%	67.86%	7.61%
LightGBM	72.75%	68.25%	64.43%	59.34%	13.41%
RF	67.74%	65.11%	62.27%	58.57%	9.17%
ROF-LightGBM (PCA)	74.28%	71.73%	69.82%	64.22%	10.06%
ROF-LightGBM (PCA, L = 4)	77.54%	75.76%	71.70%	67.61%	9.93%
ROF-LightGBM (PCA, L = 100)	76.78%	75.93%	74.48%	72.36%	4.42%
ROF-LightGBM (MNF)	82.17%	78.82%	76.44%	70.03%	12.14%
ROF-LightGBM (ICA)	75.42%	72.66%	69.95%	68.91%	9.51%

**Table 9:** Classification accuracy and running time of the RoF-LightGBM model under different rotation matrix constructors in vegetation-covered area.

Model	OA	Kappa	Time
ROF-LightGBM (PCA)	40.33%	0.31	2.31 s
ROF-LightGBM (MNF)	45.55%	0.37	2.38 s
ROF-LightGBM (ICA)	41.07%	0.32	13.38 s

## Data Availability

Field survey data and geological maps are not publicly available due to their use in other ongoing experimental studies. All other raw data supporting the conclusions of this article will be made available by the authors on request.

## Abbreviations

The following abbreviations are used in this manuscript:

HSI	hyperspectral image
AI	artificial intelligence
ML	machine learning
DL	deep learning
SVM	support vector machines
KNN	k-nearest neighbors
DT	decision trees
ROF	rotation forest
RF	random forests
OOB	out-of-bag
CNN	convolutional neural networks
PCA	principal component analysis
MNF	minimum noise fraction
ICA	independent component analysis
GOSS	gradient-based one-side sampling
EFB	exclusive feature bundling
